# Single-copy nuclear genes resolve the phylogeny of the holometabolous insects

**DOI:** 10.1186/1741-7007-7-34

**Published:** 2009-06-24

**Authors:** Brian M Wiegmann, Michelle D Trautwein, Jung-Wook Kim, Brian K Cassel, Matthew A Bertone, Shaun L Winterton, David K Yeates

**Affiliations:** 1 Department of Entomology, North Carolina State University, Raleigh, North Carolina, USA; 2 School of Biological Sciences, University of Queensland, St Lucia, Queensland, Australia; 3 Commonwealth Scientific and Research Organization – Entomology, Canberra, Australian Capital Territory, Australia

## Abstract

**Background:**

Evolutionary relationships among the 11 extant orders of insects that undergo complete metamorphosis, called Holometabola, remain either unresolved or contentious, but are extremely important as a context for accurate comparative biology of insect model organisms. The most phylogenetically enigmatic holometabolan insects are Strepsiptera or twisted wing parasites, whose evolutionary relationship to any other insect order is unconfirmed. They have been controversially proposed as the closest relatives of the flies, based on rDNA, and a possible homeotic transformation in the common ancestor of both groups that would make the reduced forewings of Strepsiptera homologous to the reduced hindwings of Diptera. Here we present evidence from nucleotide sequences of six single-copy nuclear protein coding genes used to reconstruct phylogenetic relationships and estimate evolutionary divergence times for all holometabolan orders.

**Results:**

Our results strongly support Hymenoptera as the earliest branching holometabolan lineage, the monophyly of the extant orders, including the fleas, and traditionally recognized groupings of Neuropteroidea and Mecopterida. Most significantly, we find strong support for a close relationship between Coleoptera (beetles) and Strepsiptera, a previously proposed, but analytically controversial relationship. Exploratory analyses reveal that this relationship cannot be explained by long-branch attraction or other systematic biases. Bayesian divergence times analysis, with reference to specific fossil constraints, places the origin of Holometabola in the Carboniferous (355 Ma), a date significantly older than previous paleontological and morphological phylogenetic reconstructions. The origin and diversification of most extant insect orders began in the Triassic, but flourished in the Jurassic, with multiple adaptive radiations producing the astounding diversity of insect species for which these groups are so well known.

**Conclusion:**

These findings provide the most complete evolutionary framework for future comparative studies on holometabolous model organisms and contribute strong evidence for the resolution of the 'Strepsiptera problem', a long-standing and hotly debated issue in insect phylogenetics.

## Background

Insects that undergo complete metamorphosis, collectively known as Holometabola, represent the vast majority of animal life on Earth. There are close to 1 million named species of insects [1], and the most reliable estimates suggest that the global total is between 5 and 10 million species [2,3]. Holometabola are by far the most successful group of insects, and comprise just over 80% of the named species. As far as we know, this represents about 50% of all animal diversity [4]. Understanding the relationships of the major lineages, or orders, of holometabolous insects has been a great challenge, not least because of their megadiversity, and represents one of the truly significant challenges of systematic biology.

The life history of holometabolous insects is divided into discrete developmental stages, including a distinct larval (feeding) and pupal (quiescent) stage. Most of the species-richness of this group is found in the four largest orders of insects: Coleoptera (beetles), Hymenoptera (bees, ants, and wasps), Diptera (true flies), and Lepidoptera (moths and butterflies), in addition to seven smaller orders: Neuroptera (lacewings), Megaloptera (dobsonflies and alderflies), Raphidioptera (snakeflies), Trichoptera (caddisflies), Mecoptera (scorpionflies), Siphonaptera (fleas), and Strepsiptera (twisted-wing parasites). Important model species such as *Drosophila melanogaster*, *Apis mellifera *(honey bee), *Bombyx mori *(silkworm) and *Tribolium castaneum *(flour beetle) are members of Holometabola and understanding evolutionary relationships within this diverse insect radiation is increasingly critical for comparative studies in genomics, development, and evolutionary biology.

The monophyly of the orders included in Holometabola (also known as the Endopterygota) is well established, with the exception of Mecoptera, which in some molecular analyses is rendered paraphyletic due to the inclusion of the fleas [5,6]. There is, however, less unanimity regarding the relationships between the orders. Traditional morphological hypotheses and emerging molecular results have converged on the division of Holometabola into two major lineages, Neuropteroidea, which includes Coleoptera + Neuropterida (Neuroptera, Megaloptera, and Raphidioptera), and Mecopterida (= Panorpida), including Lepidoptera, Trichoptera, Diptera, Mecoptera, and Siphonaptera [7,8]. Evidence on holometabolan phylogeny is both limited and controversial. Identification of the earliest branching extant holometabolan lineages, and resolution of the phylogenetic positions of Hymenoptera and the unusual order Strepsiptera, remains among the most disputed issues in insect phylogeny.

Hymenoptera and Strepsiptera have been placed in various positions in the holometabolan tree, the former most often placed as sister to Mecopterida and the latter traditionally included either within, or as sister to, Coleoptera [9,10]. The consensus view is that most morphological features of Hymenoptera and Strepsiptera are too highly modified to unequivocally resolve their phylogenetic positions [11,12]. Thus the placement of these two orders will necessarily rely on the use of molecular data. However, the conflicting results of the molecular studies completed to date contribute to the indeterminate nature of their evolutionary relationships. Two recent phylogenomics projects, with limited taxon sampling but including large numbers of genes, addressed the placement of Hymenoptera; mitochondrial genomes provide evidence for a sister group relationship between Hymenoptera and Mecopterida [13], while combined analysis of 185 nuclear genes shows strong support for Hymenoptera as the earliest branching holometabolan lineage, sister to all other orders [14].

Most other molecular analyses of holometabolan phylogeny rely on ribosomal DNA, and the results have been highly dependent on taxon sampling, alignment, and method of analysis [5,15-18]. The most provocative rDNA results involve Strepsiptera, a small (600 spp.) enigmatic order that maintains a degree of phylogenetic ambiguity that is unique amongst insects. Their affinity to any other order is unconfirmed, and until relatively recently, even their inclusion in Holometabola was questioned [19,20]. Strepsipterans are endoparasites of other insects, with free-living males and eyeless, larviform, viviparous females that remain inside their host (with the exception of members of the family Mengenillidae). Ribosomal DNA analyses show support for a sister group relationship between Diptera (true flies) and Strepsiptera, united in a clade called Halteria [5,17,21-24]. Dipterans and strepsipterans both possess halteres, paired knob-like structures that are homologous to wings, although dipteran halteres are found on the third thoracic segment, in place of hindwings, while strepsipteran halteres are on the second thoracic segment, in place of forewings. The initial 18S findings implied that a homeotic mutation, similar to those documented in laboratory studies with the ultrabithorax gene (*Ubx*) in *Drosophila melanogaster *[25], could have been responsible for the differing wing arrangement found in the two orders [21]; however, no supporting genetic evidence for this transformation has since been found [8,26]. Additionally, all of the morphological characters that unite Mecopterida and Antliophora (in which Halteria would be included) are lacking or inapplicable in Strepsiptera [7,8,24]. The Halteria concept also contradicts traditional interpretations of morphological characters uniting Strepsiptera and Coleoptera based on structural modifications due to posteromotorism or hindwing-powered flight [7].

Subsequent reanalyses of 18S data along with additional ribosomal DNA sequences resulted in the 'Strepsiptera problem' becoming the best known empirical example of long-branch attraction [27-30]. In a 1999 review of holometabolan phylogeny, Kristensen [8] stated that if further evidence supports Halteria, the hypothesis will be considered one of the 'most spectacular contributions of molecular characters to systematic zoology'. No additional multi-gene phylogenetic analyses have yet been completed to address the Strepsiptera question, but three additional molecular studies, one that examined an engrailed homeobox intron [26], and two that investigated the structure and evolutionary rate dynamics of ecdysone receptor and ultraspiracle proteins [31,32], failed to find any evidence of a close relationship between Diptera and Strepsiptera.

Palaeontological and phylogenetic evidence suggest an origin for Holometabola in the late Carboniferous (318-300 Ma) [1,32,33], but definitive fossil evidence is lacking until the Permian (280 Ma), a time when most of the extant orders had their origins [1]. An insect gall, presumed to be from a member of Holometabola, has been identified from the Late Pennsylvanian (302 Ma), that if accurately diagnosed provides the earliest physical evidence of their existence [34]. A molecular analysis that relied on mitochondrial data (*cox1*) and maximum likelihood (ML) global and local molecular clocks to date the origin of the insects included both dipterans and lepidopterans, and found the origin of this taxon-limited Holometabola to be between 338 Ma and 351.4 Ma [35].

To further resolve the evolutionary relationships of Holometabola and to clarify specifically the sister group to Diptera, we provide the first phylogenetic analysis to include multiple nuclear genes and representative taxa from all 11 holometabolous orders. rRNAs analyzed in previous studies were not included specifically to avoid documented biases due to alignment, long branches, compositional bias, and the unusual, divergent nature of strepsipteran rRNA [15,36,51]. Our new molecular phylogenetic data are also used to provide divergence time estimates that reveal the range of most likely dates in earth history for the origin and subsequent diversification of the extant lineages of holometabolous insects.

## Results and discussion

**Figure 1:**
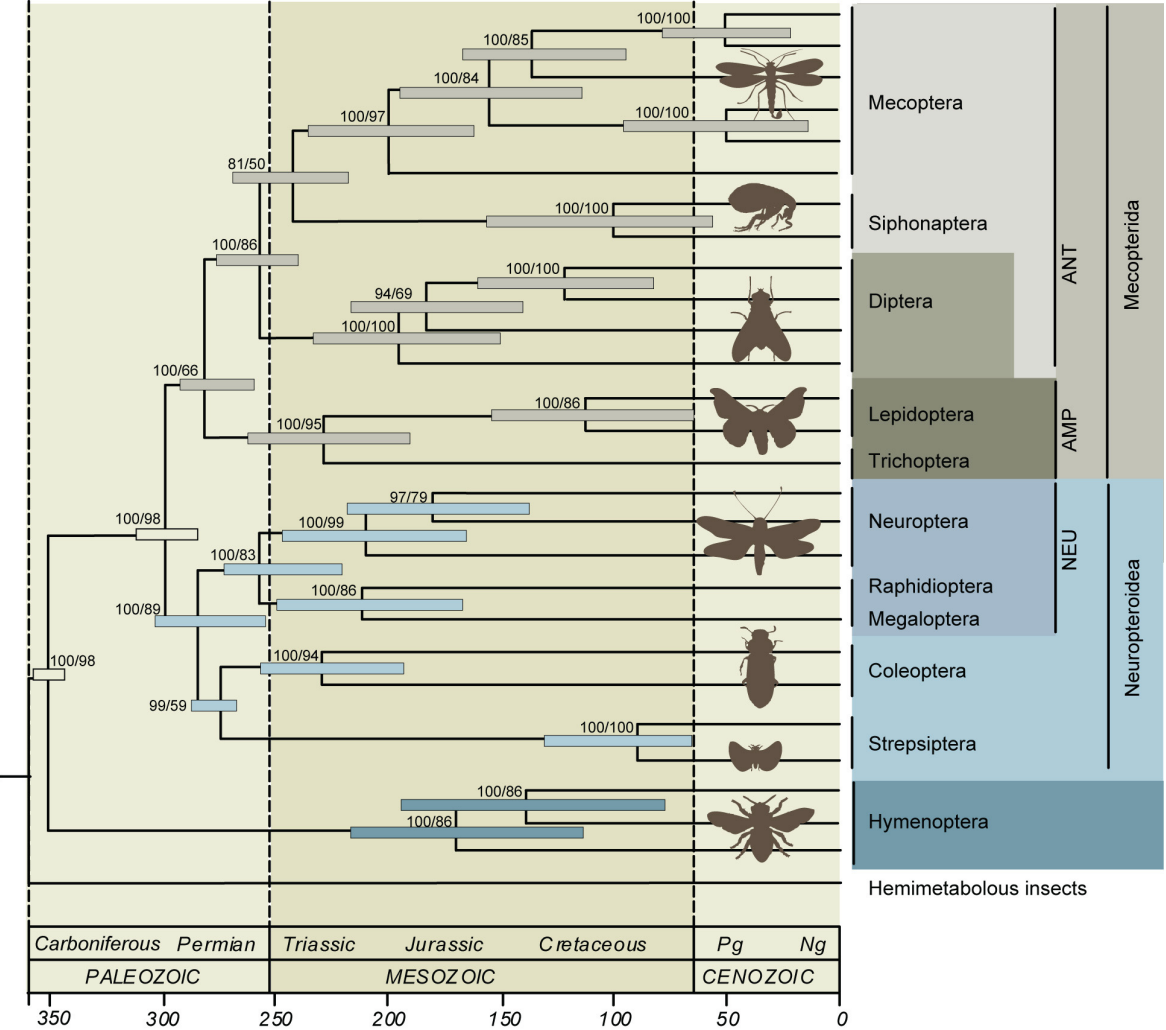
**The phylogeny of holometabolous insects with divergence time estimates**. Posterior probabilities/maximum-likelihood bootstrap values are shown at each node. Error bars reflect the 95% confidence interval surrounding each date of divergence. NEU = Neuropterida; AMP = Amphiesmenoptera; ANT = Antliophora.

**Figure 2:**
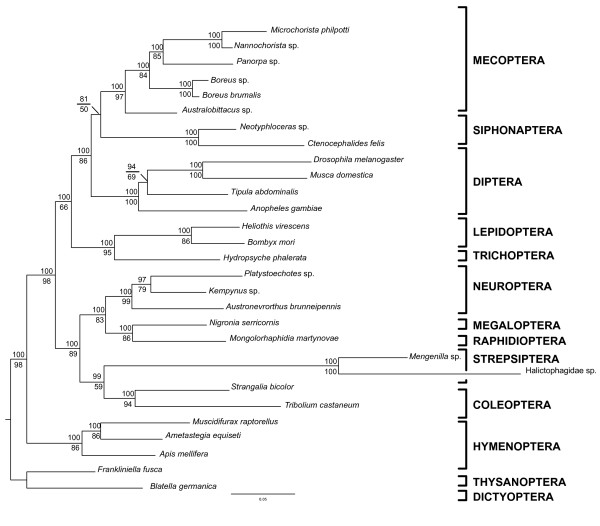
**The congruent maximum-likelihood and Bayesian topology**. Maximum-likelihood branch lengths, posterior probabilities are shown above and maximum-likelihood bootstrap values below. Although one strepsipteran in the family Halictophagidae has an exceptionally long branch, the *Tribolium *branches are only slightly longer than average.

We analyzed six nuclear genes (*AATS, CAD, TPI, SNF, PGD*, and *RNA POL II*), comprising 5736 base pairs (bp), to infer the phylogeny of 29 species representing all 11 holometabolous orders and two hemimetabolous insect outgroups (see Table 1). ML and Bayesian (BI) analyses yielded congruent trees with high posterior probabilities and mixed bootstrap values (Figures 1 and 2). All orders were found to be monophyletic, including Mecoptera with Siphonaptera as its sister group. Hymenoptera are the basal-most branching lineage, concordant with the phylogenomic findings of Savard *et al*. [14]. The enigmatic Strepsiptera are unequivocally placed as the sister group to Coleoptera, providing additional evidence for the traditional morphological placement of the twisted-wing parasites. In accordance with previous morphological and molecular hypotheses, our study finds Holometabola to be divided into two major lineages, Neuropteroidea and Mecopterida. Within these two lineages, the traditional respective supra-ordinal groupings are recovered; Neuropteroidea includes Coleoptera, Strepsiptera, and Neuropterida (Neuroptera, Megaloptera, and Raphidioptera), and Mecopterida includes Amphiesmenoptera (Lepidoptera and Trichoptera) + Antliophora (Diptera, Mecoptera, and Siphonaptera).

**Table 1:** Genes sampled for Holometabola and out-groups.

**Genes**	**Number of base pairs**	
*AATS *alanyl-tRNA synthetase	915	

*CAD *carbamoylphosphate synthase domain	2057	

*PGD *6-phosphogluconate dehydrogenase	802	

*SNF *sans fille	560	

*TPI *triosephosphate isomerase	498	

*RNA Pol II *RNA polymerase II 215 Kda subunit	899	

**Taxa**		

**Order**	**Genus species**	**Genbank number**

Dictyoptera	*Blatella germanica*	GQ265573, GQ265596 GQ265621, GQ265633 GQ265647, GQ265663

Thysanoptera	*Frankliniella fusca*	GQ265566, GQ265588 GQ265614, --------------- GQ265641, GQ265657

Hymenoptera	*Ametastegia equiseti*	GQ265565, GQ265586* GQ265587*, GQ265613 GQ265628, GQ265640 GQ265656

Hymenoptera	*Muscidifurax raptorellus*	GQ265578, GQ265604* GQ265605*, GQ265606* GQ265624, GQ265634 GQ265650, GQ265668

Hymenoptera	*Apis mellifera*	XM_395392, XM_393888, XM_625087, XM_393440, XR_014889, XM_623278

Coleoptera	*Tribolium castaneum*	XM_970534, EU677538, XM_966958, XM_963178, XM_970400, XM_968377

Coleoptera	*Strangalia bicolor*	GQ265574, GQ265599 ---------------,--------------- ---------------, GQ265664

Neuroptera	*Austronevrorthus brunneipennis*	GQ265575, GQ265600 ---------------,--------------- GQ265649, GQ265665

Neuroptera	*Kempynus *sp.	GQ265567, GQ265589 GQ265615, --------------- ---------------,----------------

Neuroptera	*Platystoechotes *sp.	GQ265568, GQ265590 GQ265616, GQ265629 GQ265642, GQ265658

Raphidioptera	*Mongoloraphidia martynovae*	---------------, GQ265597 GQ265622, --------------- ---------------,---------------

Megaloptera	*Nigronia *sp.	---------------, GQ265598 GQ265623, --------------- GQ265648, ---------------

Trichoptera	*Hydropsyche phalerata*	GQ265569, GQ265591 GQ265617, GQ265630 GQ265643, GQ265659

Lepidoptera	*Heliothis virescens*	GQ265570, GQ265592 GQ265618, --------------- GQ265644, GQ265660

Lepidoptera	*Bombyx mori*	M55993, EU032656, NM_001047060, DQ202313, NM_001126258, ---------------

Diptera	*Anopheles gambiae*	XM_318757, XM_310823, XM_313091, XM_320869, XM_321467, XM_313929

Diptera	*Tipula abdominalis*	GQ265563, GQ265584 GQ265611, GQ265626 ---------------, ---------------

Diptera	*Musca domestica*	GQ265564, GQ265585 GQ265612, GQ265627 GQ265639, ---------------

Diptera	*Drosophila melanogaster*	NM_205934, X04813, M80598, NM_078490, NM_176587, NM_078569

Strepsiptera	Halictophagidae sp.	GQ265562, GQ265583 GQ265610, --------------- GQ265638, GQ265655

Strepsiptera	*Mengenilla *sp.	---------------, GQ265580 ---------------, --------------- ---------------, GQ265651

Mecoptera	*Nannochorista *sp.	GQ265571, GQ265593* GQ265594*, GQ265619 GQ265631, GQ265645 GQ265661

Mecoptera	*Panorpa *sp.	GQ265572, GQ265595 GQ265620, GQ265632 GQ265646, GQ265662

Mecoptera	*Boreus brumalis*	GQ265576, GQ265601 ---------------, --------------- ---------------, GQ265666

Mecoptera	*Australobittacus *sp.	GQ265577, GQ265602*, GQ265603*, --------------- ---------------, GQ265667

Mecoptera	*Microchorista philpotti*	GQ265560, --------------- GQ265608, --------------- GQ265635, GQ265652

Mecoptera	*Boreus *sp.	---------------, GQ265582 ---------------,--------------- GQ265637, GQ265654

Siphonaptera	*Neotyphloceras *sp.	GQ265579, GQ265607 ---------------, --------------- ---------------, GQ265669

Siphonaptera	*Ctenocephalides felis*	GQ265561, GQ265581 GQ265609, GQ265625 GQ265636, GQ265653

Gene fragments that were unobtainable for this analysis are indicated by a horizontal line. Asterisks denote portions of CAD amplified in separate non-overlapping fragments.

It appears that the use of nuclear protein-coding genes, six in our study and 185 in Savard *et al*. [14], has brought decisive and robust results to the previously obscured phylogenetic placement of Hymenoptera. Most previous morphological hypotheses favored a sister group relationship between Hymenoptera and Mecopterida, although strong supporting evidence was lacking [7,8]. Mitochondrial genomes also favor the Hymenoptera + Mecopterida relationship, although not definitively, as the authors suggest that another 'plausible alternative placement is at the base of Holometabola' [13]. 18S rDNA paradoxically supports both previously mentioned and novel Hymenoptera hypotheses depending on alignment strategy and taxon sampling [5,15,22]. Our results constitute the tipping point of the compounding evidence (extensive sample of nuclear genes, fossil evidence, wing characters, and introns of elongation factor 1-alpha) that Hymenoptera are the earliest branching lineage of the holometabolan radiation [14,37-41].

Currently, the hypothesis that fleas are actually members of the scorpionfly order Mecoptera has gained wide acceptance [5-7]. Analyses based on morphology, ribosomal and mitochondrial DNA have strongly supported the collapse of the Siphonaptera and their inclusion within the Mecoptera as the sister group to the wingless family of snow scorpionflies, Boreidae [5-7]. Our data provide no indication of a close relationship between fleas and boreids. We found the traditional grouping of Mecoptera, with the exclusion of the fleas, to be highly supported in our analyses. No variation of taxon sampling, character inclusion, or methodology resulted in the placement of the fleas within Mecoptera. Our results suggest that the morphological characters grouping the fleas and the boreids, such as wing reduction and characters of oogenesis, be further investigated [7].

The controversial placement of Strepsiptera has been the subject of much debate, particularly in regard to whether strepsipterans are affected by a methodological artifact known as long-branch attraction (LBA). LBA is an analytical phenomenon in phylogenetic studies in which rapidly evolving sequences cluster counter to their true evolutionary history due to non-inherited similarity of rapidly accumulating mutations in independent lineages. Theoretical demonstrations of LBA identify it as a particularly difficult problem for parsimony analyses [42,43] in which the interpretation of shared derived features are maximized as the basis for explaining common ancestry [44,45]. Model-based approaches, such as ML and BI methods, make corrections for the increased chance of spurious grouping in these lineages by including information about the probability of specific changes along a branch of the tree into the analysis. However, molecular models are still widely considered to be under-developed and model-based methods can still be subject to long-branch grouping errors due to the unpredictability of evolutionary rates [46-48].

Halteria, as supported by 18S rDNA, is often cited as the first empirical evidence for LBA and initiated the development and use of parametric simulation as a statistical test for detecting LBA [28]. Both flies and strepsipterans have exhibited 'long' branches in previous 18S analyses. Similarly, in our current study one strepsipteran has a uniquely long branch, and the taxon with the next longest branch is the coleopteran *Tribolium*. To address the possibility that in our analyses the Strepsiptera + Coleoptera relationship is a spurious artifact due to LBA, we thoroughly examined our data and modified our analyses to detect and potentially rectify effects of LBA.

Although LBA is a well-documented phenomenon, its precise detection is a challenge [28,29]. Currently, the retrieval of conflicting results from maximum parsimony (MP) and ML, parametric simulation, and the visualization of conflict in a dataset can all provide suggestive evidence that LBA may be affecting an analysis [48,49]. Our parsimony trees agree with the topology generated by both ML and BI, a finding not suggestive of LBA.

Parametric simulation, a method developed by Huelsenbeck [29] to test the rDNA-based Halteria findings for LBA, can provide statistical support that branches are long enough to attract. In a procedure similar to a parametric bootstrap, simulated datasets are generated according to a tree in which taxa with elevated rates of evolution are separated in the topology; in this case, the strepsipterans are separated from the coleopterans and constrained to the base of Holometabola. The simulated datasets are then analyzed to determine whether the putative long-branched taxa will cluster counter to their placement in the tree on which the data were simulated. If Strepsiptera and Coleoptera consistently form a clade in analyses of the simulated datasets, we would conclude that grouping to be the result of LBA. None of our 100 ML analyses of the simulated data resulted in the attraction of long-branched strepsipterans and coleopterans to each other. This finding signifies that in our dataset, in contrast to the original rDNA data, there is no statistical evidence to suggest that the rates of evolution in the strepsipteran and coleopteran branches are sufficiently elevated to attract each other, counter to their accurate (simulated) evolutionary placement.

**Figure 3:**
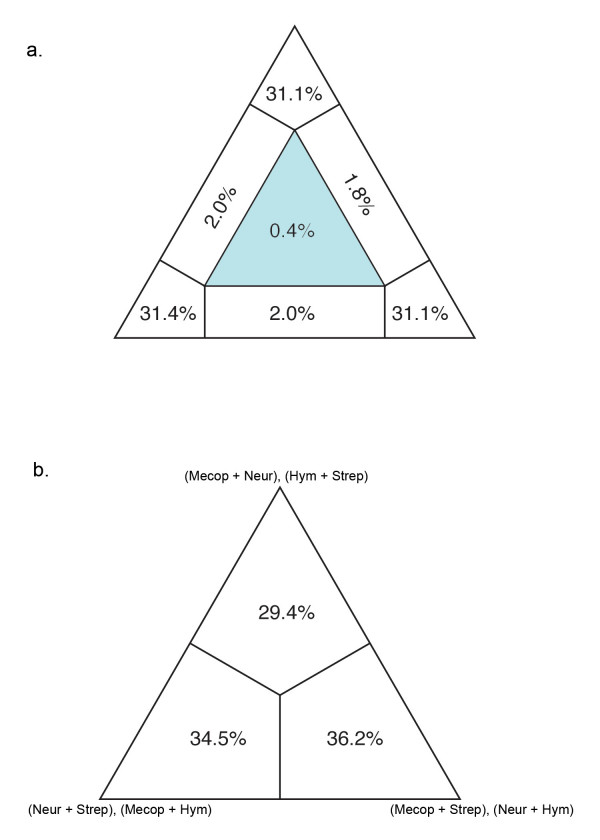
**Conflict visualization using likelihood mapping in Tree Puzzle**. **(a) **The tips of the triangle are considered 'basins of attraction' that contain the likelihoods of the percentage of quartets that are fully resolved. The center of the triangle represents the percentage (0.5%) of quartets that are unresolved; 0.4% indicated that there is not substantial conflict within our dataset [64]. (**b) **Four-cluster likelihood mapping analysis of Mecopterida, Neuropteroidea, Strepsiptera, and Hymenoptera indicates there is conflicting data supporting the affinity of Strepsiptera to each of these three groups.

**Figure 4:**
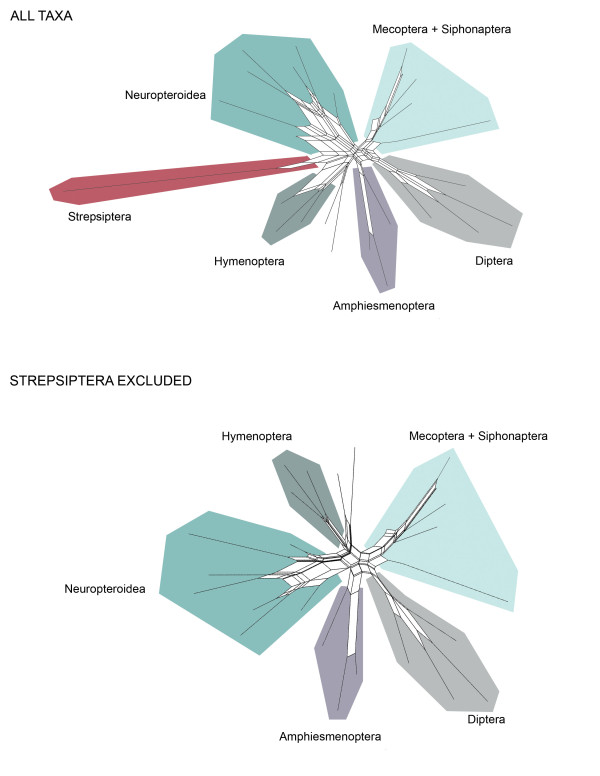
**Neighbor-nets showing conflicting splits when all taxa are included compared with when Strepsiptera are excluded**. The decreased level of conflict in the dataset exhibited when the fast-evolving Strepsiptera are excluded may be considered indicative of long-branch attraction.

In contrast to other methods that are implemented post-analysis, visualizing conflict in a dataset can be used to identify the potential for LBA prior to analysis [50]. A dataset likely to be affected by LBA should exhibit conflicting signal supporting both the artifactual relationship and the actual evolutionary relationship. We utilized two visualization methods, likelihood mapping and neighbor-nets, and our results were not definitive. Likelihood mapping, a quartet puzzling method, showed little conflict (revealed by only 0.4% of unresolved quartets while 10% to 15% is considered high) (Figure 3a). However, our neighbor-net analysis, a network showing all compatible and incompatible splits, did show conflicting signal throughout our dataset (Figure 4). The conflicting splits exist across many regions of the tree, not just regarding Strepsiptera, indicating that there is no reason to suspect LBA in regards to Strepsiptera more than other clades. Yet when a network including Strepsiptera is directly compared with a network with Strepsiptera excluded, it is evident that the conflict in this dataset is substantially alleviated by the absence of the strepsipterans, particularly in respect to the reticulation at the base of Diptera. This is not a clear sign of LBA, but it does suggest that there is conflicting support for the placement of Strepsiptera and their relationship to Diptera.

To explore further the potential for LBA identified by the neighbor-net, we utilized a four- cluster likelihood mapping analysis to again visualize the degree of conflicting signal regarding the placement of Strepsiptera. We divided the taxa into four clusters: (1) Neuropteroidea (which includes Coleoptera); (2) Mecopterida (which includes Diptera); (3) Hymenoptera; (4) Strepsiptera. The possible relationships between these four clades generate three possible topologies, each represented by a tip of the triangle. This quartet puzzling method plots the probability of each possible quartet closest to the topology that it favors. Each region of the triangle or 'basin of attraction' contains a percentage of quartets that support a particular topology. This analysis again reveals the conflicting signal in our dataset and shows that we have signal supporting all three hypotheses regarding the placement of Strepsiptera, with slightly more support in this analysis for a close relationship of Strepsiptera and Mecopterida (including the flies) (Figure 3b).

Though our concatenated dataset clearly results in the placement of Strepsiptera with Coleoptera in MP, ML, and BI, there is evidence that some signal supports a closer relationship between Strepsiptera and Diptera. To determine the source of this conflicting signal, we examined ML analyses of the six individual gene trees. Data contributing phylogenetic information for the placement of Strepsiptera is available for five out of six genes, and three out of those five genes place Strepsiptera within the close vicinity of Coleoptera or Neuropterida. The gene tree for *CAD*, however, recovers Halteria, with Strepsiptera as the sister group to Diptera. At 2000 bp, *CAD *is the longest gene in the dataset and in recent years has become a staple for resolving Mesozoic-age divergences among flies. The topology of the *CAD *ML tree reveals that Diptera and Strepsiptera all have the longest branches in the tree, similar to the initial 18S findings, suggesting the possibility that LBA may play a role in the *CAD *recovery of Halteria. It has been hypothesized that Diptera have experienced accelerated evolution in comparison to other insects [52], and by observing their long branches in various datasets we can surmise that Strepsiptera may have as well. Rapid evolution in specific loci, such as 18S and *CAD*, could lead to LBA and the erroneous grouping of Diptera and Strepsiptera. The reliance on a single locus for phylogenetic resolution, though useful in some circumstances, can clearly result in inaccurate conclusions. No single gene in our dataset recovers our well-supported phylogeny that is congruent to morphological hypotheses. Our phylogeny relies on the concatenation of all six genes to overcome the misleading signal in *CAD *placing Strepsiptera as the sister group to Diptera.

Our findings are robust over multiple phylogenetic methods intended to counter LBA including: the removal of third positions, RY coding of first and third positions, the removal of out-groups and long branches, and the use of a conservative alignment (with fast evolving positions removed by the program Gblocks [49,50,52] (Table 2). In light of the fact that our many attempts to identify or ameliorate LBA did not result in a positive detection of LBA or a change in our results, we concluded that the Strepsiptera + Coleoptera relationship is not a clear case of systematic error due to LBA. Our study is the first to rely on multiple genes to re-address the placement of Strepsiptera and our robust findings should reignite the debate regarding the morphologically dissimilar orders Strepsiptera and Diptera as sister groups. In light of our findings, upcoming work involving much larger genomic datasets (S. Longhorn, pers. comm.), and the re-examination of existing morphological characters shared by strepsipterans and beetles [7], we anticipate that the phylogenetic placement of Strepsiptera will cease to be considered the most controversial issue in holometabolan phylogenetics.

**Table 2:** Clade recovery results from maximum-likelihood analyses with varied taxon and character inclusion used to counter long-branch attraction.

Experiment	Clade recovery							
Maximum likelihood	Coleoptera + Strepsiptera	Halteria	Basal Hymenoptera	Mecopterida	Neuropteroidea (with Strepsiptera)	Amphiesmenoptera	Antliophora	Neuropterida

Third position included	+	-	+	+	+	+	+	+

Amino acids	+	-	+	+	+	+	+	+

Strepsiptera removed	N/A	N/A	+	+	+	+	+	+

Out-groups removed	+	-	N/A	+	+	+	+	+

Third RY coded	+	-	+	+	+	+	+	+

First and third RY coded	-	-	+	+	+	+	+	+

Conservative alignment	+	-	+	+	+	+	+	+

Taxa with base composition bias excluded	-	-	+	+	+	+	+	+

These variations on taxon and character inclusion (with the exception of the inclusion of third positions) are cited as a means to rectify the effects of long-branch attraction. Clade recovery from these various methods is substantially in agreement with each other and with our final results.

We used the concatenated nucleotide sequence data for all six genes, the BI phylogenetic tree topology of Figure 1, and several fossil-based minimum age constraints to estimate divergence times for major holometabolous lineages using the relaxed clock BI method implemented in the programs Estbranches and Multidivtime [53]. Congruent to the findings of Gaunt and Miles [35], multiple nuclear genes place the origin of Holometabola around 355 Ma, within the Carboniferous, but substantially earlier than traditional estimates and older than any clearly assignable holometabolan fossil (Figure 1).

As the earliest branching lineage in the phylogeny, the Hymenoptera originate just after the mean estimated age for Holometabola. This date is considerably older than existing fossil estimates (an increasingly common feature of most molecule-based divergence time estimates), a pattern suggesting either an incomplete fossil record, biases in parameter choice, model mis-specification, or some combination of these [54]. The split between the two major sub-clades Neuropteroidea and Mecopterida took place just within the Permian (300 Ma), with the Amphiesmenoptera/Antliophora diverging at 284 Ma. The origins of the extant holometabolous orders (excluding the Hymenoptera) appear to have occurred in relatively rapid succession, with dates of origin falling in the range of approximately 274 Ma to 213 Ma; the earliest divergences were the Coleoptera/Strepsiptera (274 Ma), while the most recent were the Raphidioptera and Megaloptera, splitting at 213 Ma. According to current evidence, Diptera and Mecoptera + Siphonaptera last shared a recent common ancestor approximately 256 Ma. Though some of our findings for the mean age of origin do not precisely correspond with traditional ages based on fossils, most of the published fossil-based values do fall within the 95% posterior probability range interval for our molecule-based estimates. Additionally, the insect fossil record is currently dramatically expanding [1,55,56], and thus, better fossil calibrations coupled with larger samples of genes and taxa, as well as improved analytical methods, should continue to sharpen divergence time estimates for the major holometabolous clades.

Molecular divergence time estimates and fossils agree that Holometabola had its origins within the Paleozoic, most likely in the late Carboniferous. The subsequent origins of the extant orders (excluding Hymenoptera) took place primarily within the Triassic, with primary splits occurring at the end of the Permian, and with the crown group diversification of many orders beginning in the early Jurassic. Most explanations for the enormous species diversity of holometabolous insect clades that have dominated the earth's terrestrial ecosystems since the Jurassic feature 'key innovations', such as adaptations associated with feeding on vascular plants, separation of adult, larval, and pupal stages, or morphological developments like the 'wasp waist', beetle elytron, and fly puparium [8,57-59]. Ultimately, these disparate adaptations seem to have had similar macro-evolutionary effects repeated widely across holometabolous groups; they allowed specific clades to exploit the resources provided by an increasingly complex environment and rapidly speciate in an expanding arena of biological interactions, undoubtedly propelled in many cases by flowering plant diversification [57,58,60]. The testing of key innovation hypotheses to explain the prodigious diversity of holometabolous insects remains a major task of insect phylogenetic research [33,59,61], but extreme diversity has made it difficult to resolve phylogenetic relationships among the major lineages. Consequently, conflicting lines of evidence will continue to make holometabolan phylogeny one of the most important, and revisited questions in insect phylogenetics.

## Conclusion

A new and taxonomically complete phylogenetic hypothesis for the relationships among holometabolous insect orders is presented based on six nuclear protein-coding genes. This evolutionary framework provides a critical foundation for comparative studies of insect model organisms. We have also added to the growing debate regarding the phylogenetic placement of Strepsiptera by providing the first molecular evidence that they are close relatives of beetles. The extensive analyses confirmed that the placement of Strepsiptera is not a methodological artifact, as previously proposed in other studies. Divergence time estimates show that the Holometabola emerged in the Carboniferous, while the spectacular radiations of the primary groups that exist today, wasps and bees, beetles, butterflies and flies, occurred in the Triassic.

## Methods

### Taxa sampled, DNA extraction, amplification and sequencing

A total of 29 taxa representing the 11 holometabolous orders and two hemimetabolous out-groups were sampled for sequence data from six nuclear protein-coding genes: *CAD*, *AATS*, *TPI*, *RNA POL II*, *SNF*, and *PGD *(Table 1). Taxonomic information and Genbank accession numbers are available in Table 1 and nucleotide alignments are deposited in TreeBase . Sequence alignments and trees are available for download from Treebase.org. Genomic DNA was extracted using the DNeasy DNA extraction kit and the RNA extraction kit (real time – polymerase chain reaction (PCR)) (QIAGEN Inc., Valencia, CA, USA). The standard protocol was altered by extending the amount of time the specimen was in the proteinase K solution to 2 days in order to allow enzymes to penetrate the cuticle without grinding the specimen. Final elution was reduced to 30 μl to avoid diluting the DNA solution. Genes were amplified and sequenced using degenerate primers designed by Moulton [62] for *CAD *and by JWK for the remaining five genes. PCR parameters varied for the six genes, but followed typical three-step reaction protocols (available on request from BMW). PCR products were extracted from agarose gels and purified with the Qiaquick Gel Extraction kit (Qiagen, Santa Clara, CA, USA). Big Dye Sequencing kits (Applied Biosystems, Foster City, CA, USA) were used for sequencing reactions and sequencing was completed at the North Carolina State University, Genomic Sciences Laboratory. Sequences were assembled and edited using Sequencher 4.1 (Gene Codes Corp., Ann Arbor, MI, USA). Alignment was carried out manually according to the amino acid translation using Se-Al 2.0 [63]. Introns and other positions of ambiguous alignment were removed from the analysis. To detect existing base compositional bias, a chi-square test of homogeneity of base frequencies across taxa was performed for the concatenated dataset using Tree Puzzle [64].

### Phylogenetic analyses

MP, ML, and BI analyses were completed with all positions included, third positions excluded, third positions RY coded (purine/pyrimidine coding), first and third positions RY coded, as amino acids, and of each independent gene with the third positions removed. In addition, a conservative alignment was generated in the program GBlocks, which identifies and removes areas of ambiguous alignment from the dataset [52] and analyzed with MP, ML, and BI. The following exploratory analyses with adjusted taxon sampling were completed with third positions removed: the removal of taxa with base composition bias, strepsipterans removed, coleopterans removed, and out-groups removed. These variations on character and taxon inclusion have all been suggested as means to rectify LBA [49,65].

### MP analyses

MP analyses were done using Paup* 4.0b10 [66]. Heuristic searches with tree bisection-reconnection branch swapping and 100 random addition replicates were completed to find the shortest trees. Node support was obtained by acquiring bootstrap values from heuristic searches of 500 re-sampled datasets and 10 random addition replicates.

### BI analyses

An appropriate model of nucleotide evolution, in this case GTR + I + G, was chosen by using Mr.Modeltest [67]. Using MrBayes [68,69], analyses were conducted for 5 million generations, trees sampled every 1000, with the first 25% discarded as burn-in. For nucleotide analyses, the model GTR + I + G was used with each gene treated as a separate partition; however, when third positions were included, each codon position was treated as a separate partition. For amino acid analyses, the WAG model [70] and a mixed model [71] were used, with each gene treated as a separate partition.

### ML analyses

ML analyses were performed using Garli [72] with a GTR + I + G model for nucleotides and the WAG model for amino acids. To obtain bootstrap values, 500 bootstrap replicates were performed.

### Conflict visualization

In order to visualize conflicting phylogenetic signal in our dataset, likelihood mapping and four-cluster likelihood mapping analyses were completed using the program Tree Puzzle [64]. Analyses of 10,000 quartets were completed using quartet sampling and a neighbor-joining tree with exact parameter estimates and a GTR + I + G model of substitution. To generate neighbor-nets, we analyzed a Paup* [66] generated matrix of ML inferred distances in the program SplitsTree [73]. Neighbor-nets were generated with all taxa included and with Strepsiptera excluded.

### Parametric simulation

In an effort to statistically determine whether our recovery of the Strepsiptera/Coleoptera clade was the result of LBA, we carried out a parametric simulation similar to that described by Huelsenbeck [29]. First, an input tree topology was constructed on which Coleoptera and Strepsiptera were separated (the strepsipterans were constrained to group at the base of Holometabola). Branch lengths, substitution rates, base frequencies, and gamma parameters for both first and second positions independently were calculated using Paup* 4.0b10 [66]. Using the program Mesquite v. 2.5 [74], 100 individual datasets of 1912 bp were simulated according to the constraint tree and with ML branch lengths and model parameters for both positions 1 and 2 of our empirical data. These position 1 and 2 datasets were then concatenated, resulting in 100 datasets of 3824 bp. The 100 datasets were analyzed using Garli [72]. To determine if, and how frequently, the strepsipterans grouped with the beetles, a consensus network showing all splits in the 100 resulting trees was generated in SplitsTree [73].

### Divergence time estimation

Divergence time estimates were calculated using the relaxed-clock BI MCMC method implemented in the program Multidivtime [53,75]. Inputs to the program include our dataset of six concatenated nucleotide gene sequences (excluding third position sites), the tree topology of Figure 1, and empirical model parameters and ML branch lengths calculated in the BASEML routine of the program PAML 3.4 [53,76]. To better delimit the search space and constrain ages based on several known fossils, we bounded the holometabolan root age to fall between 360 Ma (maximum age) or the approximate age of the Neoptera (a group that includes the majority of winged insects), and 280 Ma, a hypothetical minimum age for Holometabola [1]. Four additional minimum ages were set for lineages represented in the tree based on firmly established fossils: 220 Ma for Mecoptera (*Thaumatomerope neuropteroides*) [77], 227 Ma for Diptera (*Grauvogelia arzvilleriana*) [78], and 270 Ma for Coleoptera (*Sylvacoleus sharovi*) [77]. All other analysis conditions were identical to those used in Wiegmann *et al*. [75]. The effects of model prior designations on age estimates were investigated using runs that exclude the nucleotide data [53]. For analyses that included the data, multiple independent runs of the Markov chain from differing starting conditions were carried out to confirm convergence and robustness of estimated ages and rates [54].

## Abbreviations

BI: Bayesian; bp: base pairs; LBA: long-branch attraction; ML: maximum likelihood; MP: maximum parsimony; PCR: polymerase chain reaction.

## Authors' contributions

BMW, JWK, MDT, MAB, SLW, and BKC conceived and designed the experiments. JWK and BKC designed oligonucleotides and collected data. MDT, JWK, and BMW performed the experiments and analyzed the data. SLW, MAB, DKY, and BKC contributed specimens/reagents/materials/analysis tools. MDT, BMW, and DKY wrote the paper.
